# Fabrication of Nanopillar Crystalline ITO Thin Films with High Transmittance and IR Reflectance by RF Magnetron Sputtering

**DOI:** 10.3390/ma12060958

**Published:** 2019-03-22

**Authors:** Ling Dong, Guisheng Zhu, Huarui Xu, Xupeng Jiang, Xiuyun Zhang, Yunyun Zhao, Dongliang Yan, Le Yuan, Aibing Yu

**Affiliations:** 1 Guangxi Key Laboratory of Information Materials, Guilin University of Electronic Science and Technology, Guilin 541004, China; 18589941163@163.com (L.D.); xuhuarui@163.com (H.X.); 18593275673@163.com (X.J.); xyz_guet@163.com (X.Z.); yunyzhao@163.com (Y.Z.); dlyan@guet.edu.cn (D.Y.); 2 School of Materials Science and Engineering, Xihua University, Chengdu 610039, China; yuanle.cn@gmail.com; 3 ARC Hub for Computational Particle Technology, Monash University, Clayton, Victoria 3800, Australia; aibingyu0325@163.com

**Keywords:** indium tin oxide thin film, RF magnetron sputtering, nanopillar crystalline, transmittance, IR reflectance

## Abstract

Nanopillar crystalline indium tin oxide (ITO) thin films were deposited on soda-lime glass substrates by radio frequency (RF) magnetron sputtering under the power levels of 100 W, 150 W, 200 W and 250 W. The preparation process of thin films is divided into two steps, firstly, sputtering a very thin and granular crystalline film at the bottom, and then sputtering a nanopillar crystalline film above the bottom film. The structure, morphology, optical and electrical properties of the nanopillar crystalline ITO thin films were investigated. From X-ray diffraction (XRD) analysis, the nanopillar crystalline thin films shows (400) preferred orientation. Due to the effect of the bottom granular grains, the crystallinity of the nanopillar crystals on the upper layer was greatly improved. The nanopillar crystalline ITO thin films exhibited excellent electrical properties, enhanced visible light transmittance and a highly infrared reflectivity in the mid-infrared region. It is noted that the thin film deposited at 200 W showed the best combination of optical and electrical performance, with resistivity of 1.44 × 10^−4^ Ω cm, average transmittance of 88.49% (with a film thickness of 1031 nm) and IR reflectivity reaching 89.18%.

## 1. Introduction

Indium tin oxide (ITO) thin film is a kind of degenerate semiconductor material with excellent photoelectric properties, which is widely used as flat panel displays [1], touch screen panels [2], light emitting diodes [3], solar cells [4,5], and infrared stealth coating [6]. ITO thin films have been prepared by various coating techniques, such as spray pyrolysis [5], electron beam evaporation [7], direct current (DC) magnetron sputtering [8,9], radio frequency (RF) sputtering [10,11] and sol-gel [12]. In particular, RF/DC sputtering has the advantages of producing thin films with superior crystalline quality compared with other preparation methods. 

In this study, we mainly focused on the application of ITO thin films in the infrared stealth field. ITO thin films applied in the infrared stealth field must possess excellent conductivity, a high visible light transmittance, and a desirable IR reflectivity. In addition, the research of Xuesong Yin [13] reveals that the thickness of ITO thin films applied to infrared stealth contexts are close to the micron level or even greater than that. Generally, the conductivity of ITO thin films tends to be better with the increase of film thickness, but the visible light transmittance has a significant reduction with the increase of film thickness [14,15]. Based on the Drude theory [16], the IR reflectance of ITO thin films can be enhanced by increasing the conductivity; therefore resolving the contradiction between the visible light transmittance and IR reflectivity of ITO thin films is the focus of this study. K. Aijo John [10] has pointed out that the ITO thin films with (400) preferred orientation exhibit outstanding performance in conducting behavior. Kim [17] has also shown the ITO thin films with (400) preferred orientation have higher carrier concentrations than the ITO thin films with (222) preferred orientation. It was also reported that the transmittance of ITO thin film is closely related to its crystallinity [18]. Moreover, in terms of the transparency of ITO thin films, Hoo Keun Park [19] has reported a nanorod ITO thin film which exhibited excellent transparency. 

**Figure 1:**
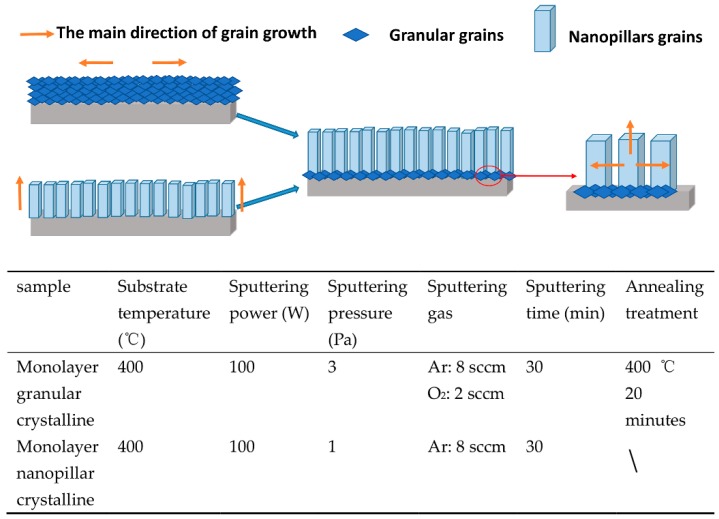
Schematic diagram of multiple structure nanopillar crystalline film, the preparation parameters of monolayer granular crystalline film and monolayer nanopillar crystalline film.

In order to fabricate ITO thin films with excellent conductivity, a high visible light transmittance, and an ideal IR reflectance, a two-step sputtering method was carried out to deposit a very thin and granular crystalline film at the bottom combining with nanopillar crystalline film on the upper. As illustrated in Figure 1, the granular grains at the bottom are attached around the nanopillar grains, due to the different growth orientation between granular grains and nanopillar grains, which has enlarged the space for lateral growth of the nanopillar crystals, thus the nanopillar crystalline thin films grow better. The structure, morphology, and photoelectric properties of the nanopillar crystalline ITO thin films deposited at different sputtering powers are discussed in detail. 

## 2. Materials and Method

The ITO thin films were deposited on 1.1 mm thick soda-lime glass substrate (50 mm × 50 mm × 1.1 mm) using a JPG-560C12 (SKY Technology Development Limited Company of Chinese Academy of Sciences, Shenyang, China) RF magnetron sputtering system with an ITO ceramic target (In_2_O_3_, 90 wt % and SnO_2_, 10 wt %, 99.99% purity, diameter of 60 mm and thickness of 5 mm, Guangxi Huaxi Group Co., Ltd., Liuzhou, China). Cleaning treatment of soda-lime glass substrates was carried out by dipping in acetone, absolute ethyl alcohol, and deionized water, ultrasonic treatment for 10 min orderly, then rinsing in absolute ethyl alcohol and drying in nitrogen for use. 

The distance between the substrate and the target was set to 70 mm. Before deposition, the chamber pressure was vacuumed to 2 × 10^−4^ Pa. Firstly, a granular crystalline thin film was deposited at 100 W with Argon and Oxygen (with a flow rate of 8 and 2 sccm respectively), and the working gas pressure was set to 3 Pa. The deposition process was carried out at 400 °C for 1 min. After deposition, the film was annealed for 20 min in the chamber. To fabricate a nanopillar crystalline thin film above the granular grains, Argon was injected into the chamber at a flow rate of 8 sccm, and the working gas pressure was maintained at 1 Pa. The deposition was processed at 400 °C for 30 min. The multiple structure nanopillar crystalline ITO thin films were deposited at 100 W, 150 W, 200 W, and 250 W. 

X-ray diffraction (XRD-D8 Advance, Brucker Inc., Hamburg, Germany, a wavelength of 0.15405 nm) was used to characterize the crystalline structure and lattice parameters of the ITO thin films. The surface and cross-sectional morphologies of the ITO thin films were investigated using field emission scanning electron microscope (FE-SEM, FEI Tecnai-450, Hillsboro, OR, USA). The surface 2D morphologies of the ITO thin films were analyzed by atomic force microscope (AFM, MultiMode 8, Brucker). Electrical resistivity, carrier concentration, and Hall mobility were measured by Hall measurement system (Nanometric HL5500PC, Shenzhen Aonei Electronics Co., Ltd., Shenzhen, China). Transmission spectra were tested by ultraviolet-visible spectrophotometer (U-4100 type Hitachi, Tokyo, Japan). The IR reflectance spectrum was measured with a Fourier transform infrared spectrometer (Tensor27, Bruker) with an integrating sphere attachment. 

## 3. Results and Discussion

### 3.1. Structure and Morphology Analysis 

#### 3.1.1. XRD Analysis 

**Figure 2:**
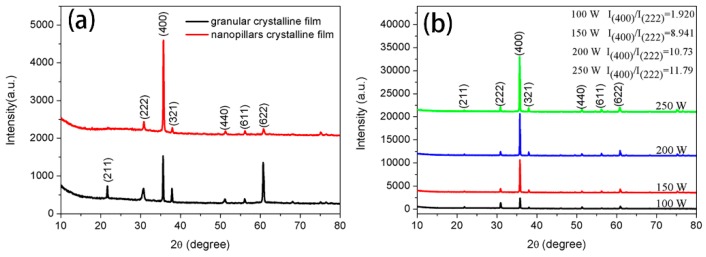
(**a**) X-ray diffraction (XRD) patterns of monolayer granular crystalline and monolayer nanopillar crystalline ITO thin film; (**b**) XRD patterns of multiple structure nanopillar crystalline indium tin oxide (ITO) thin film deposited at different powers.

Figure 2a shows the XRD results of the monolayer granular crystalline and monolayer nanopillar crystalline ITO thin film. The main diffraction peaks of the former were (400) plane and (622) plane, but the latter’s main diffraction peak was (400) plane. XRD patterns of the multiple structure nanopillar crystalline ITO thin films deposited at different powers are shown in Figure 2b. The results show that the films are cubic phases, whose diffraction peaks are in accordance with the standard JCPDS # 06-0416. 

In terms of multiple structure nanopillar crystalline film deposited at 100 W, the intensity of the (400) diffraction peak is slightly higher than that of (222) diffraction peak. This phenomenon is owing to the fact that there is a very small amount of oxygen left after the deposition of granular films. Jae-Hyung Kim [17] has verified that Oxygen addition is not conducive to the (400) preferred growth of ITO thin films. However, it is seen from the XRD patterns that the ITO thin films deposited at 150 W, 200 W, and 250 W have a sharp (400) diffraction peak. Moreover, there is a sudden increase in the intensity of (400) plane between 100 W and 150 W, which can be demonstrated by the different growth rates among grains with different preferred orientations [20,21]. The calculated results of I_(400)_/I_(222)_, presented in Figure 2b, reveal that the grains with (400) preferred orientation grows faster. Additionally, the growth mechanism of the ITO thin films with different preferred orientations has been studied by Jae-Ho Kim [14], and they believed that the (400) plane grows quickly due to its lower surface energy. 

Grain sizes of the crystallized samples are calculated using the Debye-Scherrer equation [22].

$$d=\frac{K\lambda }{\beta cos\theta }$$

where *d* is the grain size, *K* is the shape factor taken as 0.943, *λ* is the wavelength of the incident beam, *β* is the full width at half maximum and *θ* is the Bragg’s angle. The average grain size corresponding to (400) plane was calculated to be 86.70 nm, 91.74 nm, 98.50 nm and 112.2 nm for multiple structure nanopillar ITO thin films deposited at 100 W, 150 W, 200 W and 250 W respectively. The average grain size of monolayer nanopillars was calculated to be 80.49 nm. Grain sizes of the nanopillar films corresponding to (400) plane could also imply that the degree of crystallization has been improved. In the deposition process, the Ar^+^ possesses greater kinetic energy with higher bias voltage, thus contributing to the improvement of the crystallization process. 

#### 3.1.2. SEM Analysis 

**Figure 3:**
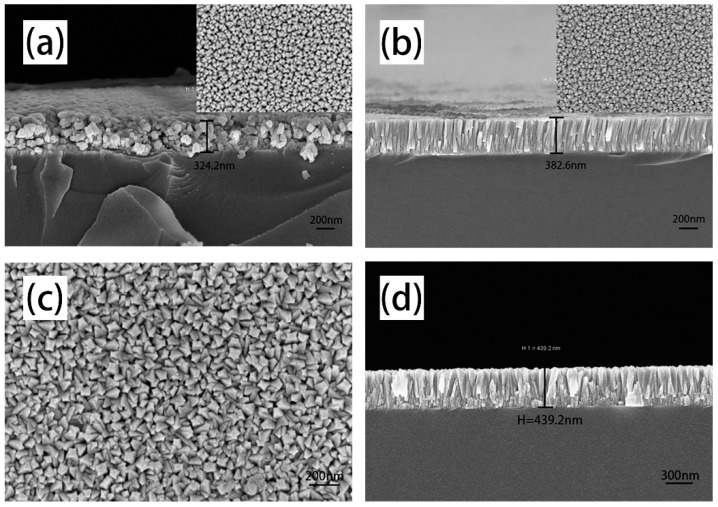
(**a**,**b**) Surface and cross-section morphology images of monolayer granular crystalline and monolayer nanopillar crystalline ITO thin film by scanning electron microscope (SEM); (**c**,**d**) Surface and cross-section SEM images of multiple structure nanopillar crystalline film deposited at 100 W.

Surface and cross-section morphology views of the ITO thin films are presented in Figure 3. Multiple structure nanopillar crystalline ITO thin films were deposited by combining the preparation process of monolayer granular crystalline film and monolayer nanopillar crystalline film. As Figure 3c,d shows, compared to monolayer granular crystalline and monolayer nanopillar crystal ITO thin film, whose cross-section and surface images are described in Figure 3a,b respectively, the multiple structure nanopillar film possesses a thin layer of granular crystallization at the bottom of the film with a more obvious nanopillar crystals above the granular grains. 

**Figure 4:**
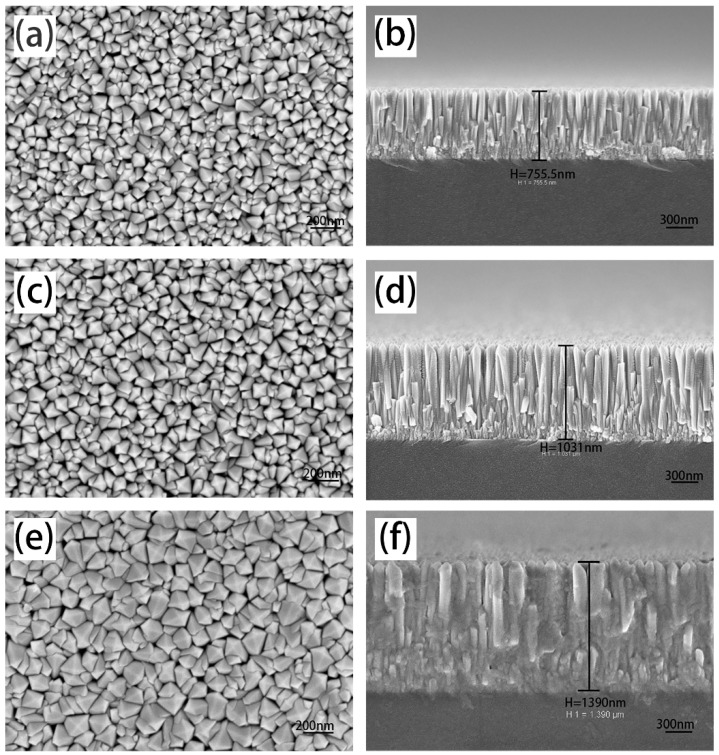
Surface and cross-section morphology images of multiple structure nanopillar crystalline films by SEM: (**a**,**b**) 150 W; (**c**,**d**) 200 W; (**e**,**f**) 250 W.

Multiple structure nanopillar crystalline film was deposited at 100 W, whose surface SEM image shows triangular and square uniform grains (shown in Figure 3c). Focus on the top-views of other samples deposited at higher sputtering powers, shown in Figure 4, the surface morphology of grains are mainly square. Furthermore, the degree of crystallization has been improved, which can also be verified from the thickness of multiple structure nanopillar films, and the thickness of the films is 439.2 nm, 755.5 nm, 1031 nm and 1390 nm for films deposited at 100 W, 150 W, 200 W, and 250 W, respectively. Simultaneously, it has been noted that the sputtering time of the bottom granular layer and upper nanopillars layer was fixed at 1 min and 30 min respectively. Sputtering rates at different sputtering powers could be approximately calculated to be 0.24, 0.41, 0.57, 0.77 nm/s. 

#### 3.1.3. AFM Analysis 

**Figure 5:**
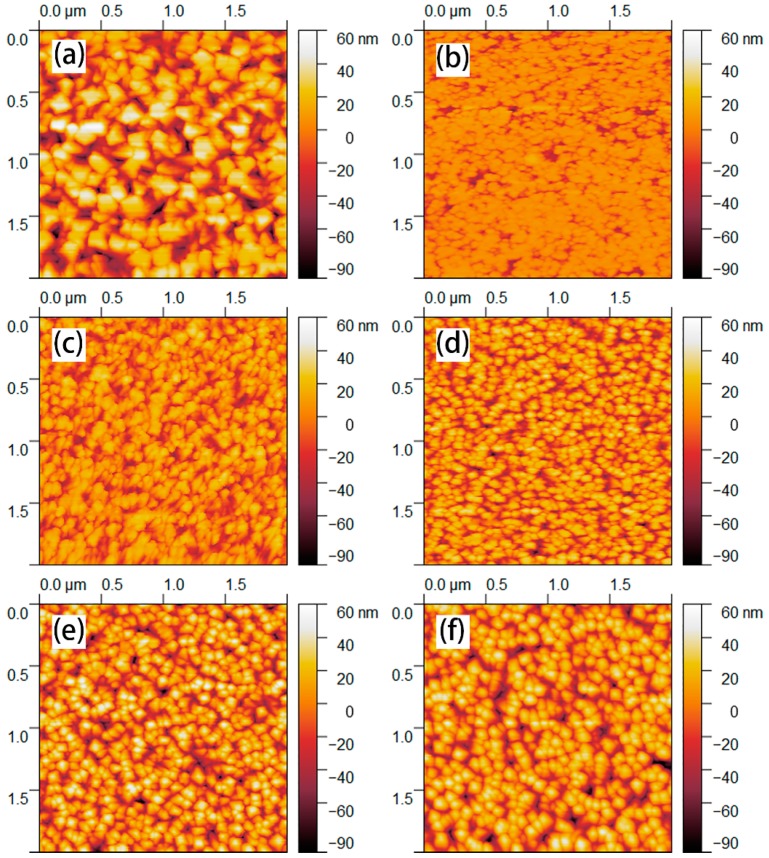
2D morphology of films by atomic force microscope (AFM) (**a**,**b**) Monolayer granular and nanopillar crystalline films; (**c**–**f**) The multiple structure nanopillar crystalline films deposited at 100 W, 150 W, 200 W and, 250 W, respectively.

Figure 5 shows the AFM images of the surface 2D morphologies of all samples. The average roughness parameter (Ra) of the monolayer granular crystalline film determined from AFM data of 2 μm × 2 μm scan area was 19.82 nm. However, as Figure 5b shows that the surface of the monolayer nanopillar crystal is smoother, with an average roughness parameter of 6.144 nm. Figure 5c displays the surface AFM image of multiple structure nanopillar crystalline ITO thin film that deposited at 100 W, whose average roughness is 13.39 nm. Combined with the SEM study results, it could be demonstrated that the two-step deposition method is applied as described in the schematic. 

In Figure 5c–f, the grain sizes and morphology are both consistent with their SEM images. The multiple structure crystalline nanopillar ITO thin films possess a sharp columnar cap, which has an advantage over the transmission of visible light compared with the relatively smooth columnar cap of monolayer nanopillar crystalline films. Average roughness parameters (Ra) are 13.93, 17.24, 21.15 and 17.03 nm for multiple structure nanopillar films deposited at 100 W, 150 W, 200 W, and 250 W, respectively, which is one to two times larger than those that have been reported [23,24]. Thus, in this study, the roughness mainly reveals the longitudinal growth trend of thin films. However, there is a decrease in surface roughness for the film deposited at 250 W. A possible explanation for this phenomenon is that the transverse growth trend of the film is larger than that of longitudinal growth. Grain size could indirectly demonstrate the transverse growth trends, besides, the grain size of the film deposited at 250 W increased remarkably. Thus, the roughness of film deposited at 250 W has fallen.

### 3.2. Electrical Properties 

**Figure 6:**
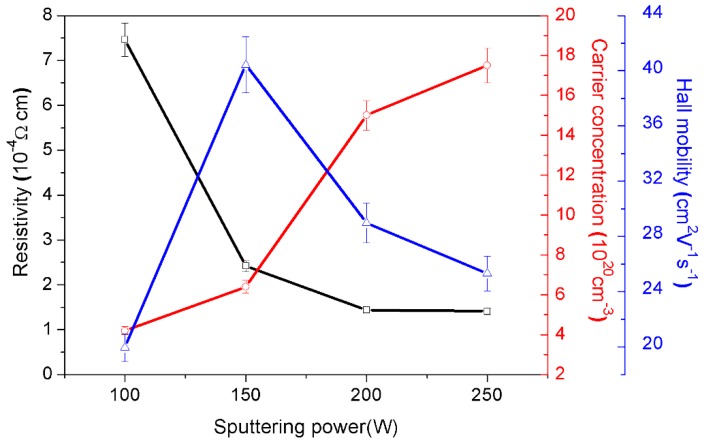
Electrical properties of multiple structure nanopillar crystalline films, including resistivity, carrier concentration, and mobility.

Electric parameters of the multiple structure nanopillar crystalline ITO thin films, including electrical resistivity, carrier concentration, and mobility, are shown in Figure 6. The measurement results are summarized in Table 1. Obviously, resistivity decreased with the increase of sputtering power from 100 W to 250 W, however, the resistivity decreased slightly when the sputtering power increased to 250 W. As shown in Table 1, the increased film thickness, caused by higher sputtering power, led to an increase in carrier concentration. 

In terms of carrier mobility, there was a sharp increase between sputtering power of 100 W and 150 W. We believe that the relatively excessive grain boundary of the film deposited at 100 W might make free carriers drop or scatter thereby showing lower mobility [15,25,26], as shown in Table 1, the carrier mobility of the film deposited at 100 W is 19.95 ± 0.997 cm^2^ V^−1^s^−1^, which is far less than that of film deposited at 150 W (40.43 ± 2.201 cm^2^ V^−1^s^−1^). 

It was found that the film deposited at 150 W possessed the highest carrier mobility, the mobility of film deposited at 200 W and 250 W decreased unexpectedly. This indicates that the damage by increased defects, causing by structure damage resulting from the increased discharge voltage and the bias voltage (shown in Table 1), will decrease the mobility. Although the carrier concentration of the thin film is increased, the rate of increase slows down. In this study, the thin film deposited at 200 W shows excellent conductivity. In our previous work [27], we prepared ITO thin films by RF sputtering at 200 W; the as-annealed film has the best resistivity of 2.08 × 10^−4^ Ω cm. Fortunately, the resistivity of multiple structure nanopillar crystalline film deposited at 200 W could be as low as 1.44 × 10^−4^ Ω cm. 

### 3.3. Optical Properties 

**Figure 7:**
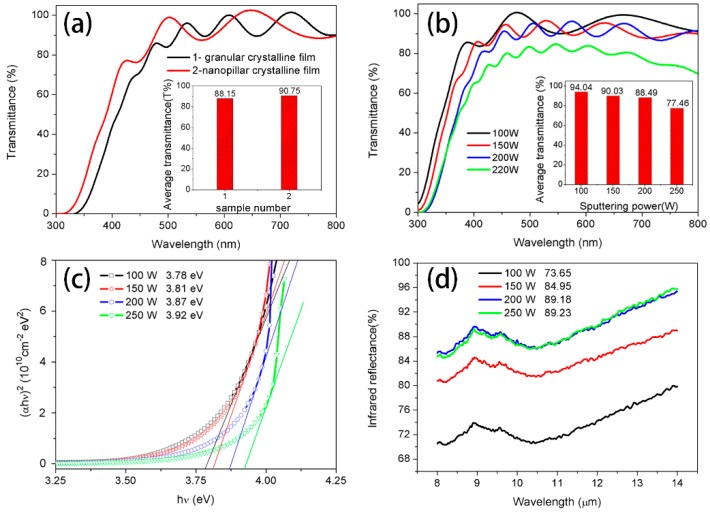
(**a**) Visible light transmittance spectra of monolayer granular and nanopillar crystalline ITO thin film; (**b**) Visible light transmittance spectra of multiple structure nanopillar ITO thin films deposited at different temperature; (**c**) Energy band gaps of the multiple structure nanopillar films; (**d**) IR reflectivity of multiple structure nanopillar films in mid-infrared region.

Figure 7a illustrates the optical transmittance spectra of the monolayer granular crystalline (with an average visible light transmittance of 88.15%) and monolayer nanopillar crystalline (with an average transmittance of 90.75%) ITO thin films. According to the results shown in Figure 7b, the multiple structure nanopillar crystalline film deposited at 100 W has a sharp higher average transmittance (94.04%) than the monolayer nanopillar crystalline film. This enhancement of optical transmission may be caused by the good crystallization of thin films. Moreover, light-trapping at the cap valley of the nanopillar crystals may reduce reflection of the light, thus leading to enhanced visible light transmittance. Increased light harvesting indicates that the films are more transparent in the visible region. Similar antireflection effects have been observed in thin films with wrinkle micro-patterns surface [28]. Chuang Wang [29] has also reported that optical transmittance may be enhanced by light-trapping at the valleys of the wrinkles.

In addition, the average transmittance decreased gradually with the increasing film thickness resulting from increased sputtering power. The increased thickness of the film may have mainly lead to a decrease in transparency. The decrease in transparency could also be attributed to free carrier absorption. Even so, compared with the research results (ITO thin film of 1000 nm with an average transmittance of 71.38%) in Reference [14], the result of this study has a clear advantage. 

The optical band gap *E_g_* can be calculated from the absorption coefficient, which can be calculated from the transmission spectra using equations [30]:

$$\alpha =\frac{1}{t}ln\left(\frac{1}{T}\right)$$

where *T* is the transmission and *t* is the thickness of the films. The optical band gap of ITO thin films can be calculated using the following equation [30]:

$$\alpha h\nu =A{\left(h\nu -E_{g}\right)}^{m}$$

where *α* is the absorption coefficient, *h* is the Planck’s constant, *ν* is the frequency of incident light, *A* is constant, *E_g_* is the energy band gap of films and *m* equals to 1/2 for direct semiconductors. The energy band gap, shown in Figure 7c, was calculated to be 3.78 eV, 3.81 eV, 3.87 eV and 3.92 eV for films deposited at 100 W, 150 W, 200 W, and 250 W, respectively. We believe that the reason for energy band gap variety is the increasing number of carriers, which lead to an increase in the Fermi level above the bottom of the conduction band, thereby causing enlargement in the optical band gap of the ITO films [15]. 

It has been reported that the IR reflective of transparent conductive thin films originated from the plasma, which was formed by a high concentration of electrons [31]. According to the Drude theory [16], the IR reflective is directly related to the carrier concentration and mobility of materials. P.K. BiswasM [32] has studied the effect of tin on IR reflectivity of indium tin oxide thin film, and found that increasing carrier concentration and Hall mobility of the samples are key factors in improving infrared reflectivity. It is well known that the relationship among resistivity, carrier concentration, and mobility could be represented by the following equation [13]:

(1) $$\rho =\frac{1}{qn\mu }$$

*ρ* is resistivity, *q* presents electron charge, *n* is carrier concentration and *μ* is mobility. According to this formula, nanopillar crystalline film with lower resistivity has a higher IR reflectivity. As Figure 7d shows, the multiple structure crystalline film deposited at 200 W and 250 W both reveal a desirable IR reflectivity.

## 4. Conclusions

Multiple structure nanopillar crystalline ITO thin films have been successfully prepared by a two-step sputtering method. This multiple crystalline nanopillar ITO thin film exhibited excellent electrical properties, enhanced visible light transmittance and a high infrared reflectivity in the mid-infrared region. Nanopillar films with different thickness deposited under different powers were discussed. It was found that the increase of sputtering power lead to the increase of discharge voltage and the bias voltage, which plays an important role in determining the photoelectric properties of the ITO thin films. The carrier mobility and visible light transmittance of the film decreased sharply when the sputtering power approached 250 W. As a result, the thin film deposited at 200 W (with a film thickness of 1031 nm) shows the best combination of optical and electrical performance, with resistivity of 1.44 × 10^−4^ Ω cm, average transmittance of 88.49% and IR reflectivity reaching 89.18%. This study provides a new idea for the application of ITO thin films in infrared stealth field. 

## Figures and Tables

**Table 1:** Discharge voltage and Bias voltage under different sputtering powers; Resistivity, carrier concentration and mobility of the multiple structure nanopillar films.

Sputtering Power (W)	Discharge Voltage (V)	Bias Voltage (V)	Resistivity (10^−4^ Ω cm)	Carrier Concentration (10^20^ cm^−3^)	Mobility (cm^2^ V^−1^ s^−1^)
100	834	176	7.46 ± 0.373	4.20 ± 0.210	19.95 ± 0.997
150	1012	235	2.42 ± 0.121	6.40 ± 0.320	40.43 ± 2.201
200	1172	290	1.44 ± 0.072	15.0 ± 0.750	28.98 ± 1.449
250	1296	339	1.41 ± 0.070	17.5 ± 0.875	25.32 ± 1.266
